# Associations between walkability and physical activity among children and adolescents: evidence from a gamified intervention

**DOI:** 10.1186/s12889-025-25317-0

**Published:** 2025-11-06

**Authors:** Laura Eipel, Paula Teich, Fabian Arntz, Daniel Scheller, Christoph Mall, Jan Schmid-Ellinger

**Affiliations:** 1 Chair of Social and Preventive Medicine, Potsdam University, Potsdam, Germany; 2 TUM School of Medicine and Health, Technical University of Munich, Munich, Germany; 3 Planersocietät, Karlsruhe, Germany

**Keywords:** Walkability index, Physical activity, Intervention, Children and adolescents, Urban design and planning

## Abstract

Children and adolescents often do not meet the WHO´s physical activity (PA) recommendations. As many of them live in urban areas, these are important spaces for PA-promotion. Objective measures such as the Walkability Index are often used to assess urban spaces in terms of their PA friendliness. However, it is unclear whether such parameters can predict PA behavior of children and adolescents. This study examines the relationships between the Walkability Index and data of the intervention “Kreuz & Quer” (K&Q), promoting PA. K&Q collected data from 9,852 children and adolescents in urban neighborhoods. PA was measured through the number of interactions at K&Q checkpoints, reflecting participants’ actively performed visits to physical locations. PA acted as the dependent variable in a linear mixed models approach. Walkability served as a fixed factor and district, season of year and intervention day as random effects. Results indicate a significant positive association between a high Walkability Index and PA levels in children and adolescents. Some of the observed variance can be explained by the random effects. There is still unexplained variance, suggesting the need to consider additional influences to explain youth PA behavior. These may include qualitative explanations to provide a holistic picture. Subjective perspectives can help create environments that are structurally conducive to walking, thereby promoting PA.

## Introduction

### Physical (in)activity in children and adolescents

Physical activity (PA) is related to multiple health benefits. Regular movement can increase physical, mental, and social health [1–3]. It can strengthen the cardiovascular system and the development of the musculoskeletal system [4, 5]. In addition, PA helps to prevent medical conditions, such as overweight and obesity, or cancer [6–8]. A sedentary lifestyle, on the contrary, has a negative impact on health [9, 10].

It is estimated that 80% of adolescents across the globe are categorized as inactive [9, 11] In Germany, only 22,4% of girls and 29,4% of boys aged three to 17 reach the recommendations of the World Health Organization (WHO) of 60 min of moderate to vigorous PA per day. Especially childhood though should be regarded as an important stage of life to provide a basis for a health-conscious behavior in later life [5, 9, 12, 13]. The number of children and adolescents who reach the WHO recommendations should certainly be higher than it currently is.

Individual PA behavior can be seen as a product of multiple interacting factors. In children and adolescents, those factors encompass demographic, individual, social, and environmental variables [14]. These environmental variables, or physical infrastructures such as parks, buildings and recreational facilities can either enable or hinder PA and encompass “settings such as the neighborhood or schools based on site-specific physical infrastructures” [15]. These settings provide spaces for both individual PA and social interaction [15]. For instance, PA is positively influenced by a residential environment that provides sufficient opportunities for PA [16]. One such opportunity is that of active travel, a factor which has the potential to influence positive changes in PA and sedentary behavior (SB) in daily life [17]. However, in terms of different modes of transport within a city, the car is the dominant mode of transport as stated in the German mobility profitability report [18]. Children up to the age of nine are heavily dependent on their parents for mobility and are driven half the time dedicated to travel, which means that children are strongly socialized by this mode of transport [18]. A study by Hillman et al. [19] is still influential in urban planning, childhood studies, and public health. It revealed a dramatic decline in the number of children permitted to travel without adult supervision (Independent Mobility [IM]). In 1971, 80% of seven- to eight-year-olds walked to school alone; by 1990, this had dropped to just 9%. The main reason parents limited their children’s mobility was fear of road traffic. Schoeppe et al. [20] found an ongoing decline in IM due to parental concerns about road safety, stranger danger, or longer travel distances to schools and recreational facilities. Han et al. [21] note, that despite an increasing awareness of the benefits of PA and IM, more parents are limiting children’s freedom. These restrictions manifest in the establishment of boundaries, time limits and spatial constraints [22, 23]. For instance, it is not permissible for children to walk or cycle without adult supervision. Furthermore, access to parks or streets in the vicinity is restricted, and children are instead encouraged to travel by car rather than walking [22, 23]. The authors posit that this creates a vicious cycle: a decline in the number of children in public space leads to less perceived safety, which further reduces autonomy. Consequently, children lose opportunities to develop spatial awareness, decision-making skills, and confidence. The authors warn that this shift is conducive to greater car dependency, reduced physical activity, and weaker community engagement [19, 20].

Active travel by foot or bike coul help to build a sense of independence, self-confidence, and concern for the environment, and plays an important role in improving the health of children and adolescents [17]. There are many approaches to the promotion of PA, of which active travel in the urban built environment is one of the most promising.

### Promotion of physical activity

Research findings underscore a complex interplay of factors influencing children´s and adolescents’ engagement in PA. Hu et al. [24] categorize influencing factors into the levels intrapersonal, interpersonal, organizational, community and public policy and thereby refer to the social ecological model (SEM) proposed by McLeroy et al. [25]. A key factor that can be categorized at the organizational level and seen as an environmental approach is the built environment. Public health research provides strong evidence that the built environment can influence overall health by promoting PA in younger populations [26, 27]. In the context of the built environment, PA pertains to various factors, including the extent of movement during walking and active travel within a neighborhood [28–30]. Associations have been observed between neighborhood design and walking and cycling for transportation [31]. Studies show that an increase in walking can be achieved through mixed-use residential, commercial, office, entertainment, and other land use [32, 33]. Hence, urban planning should focus on promoting walking as an important factor of PA for improving public health. The finding’s consistency across cities indicates the potential value of engaging the urban planning, transportation, and parks sectors in efforts to mitigate the health consequences of global physical inactivity [34].

This raises the question of how health- and walk-promoting urban design can best be achieved, particularly with the specific target group of children and adolescents in focus. In this regard, schools, their surroundings, and the immediate living environment of children and adolescents may be a particularly promising settings for interventions to promote PA [35, 36]. Kelso et al. [15] identified the neighborhood, school, and recreational environments as the settings most frequently used for PA in children and adolescents. Studies reported higher PA values for children and adolescents on streets, roads and pavements rather than in locations with green spaces, which could be due to more time being spent in built environments [15].

Additionally, participation in programs aimed at promoting PA seems to be strongly influenced by social interactions, such as those with peers and friends, referring to the interpersonal level [24]. According to this, play, especially for children, represents a relevant factor in getting involved in neighborhood PA. In this regard, gamified scenarios appear to be beneficial for this target group [37, 38]. In these cases, traditional button presses in sedentary video games are replaced with gross motor movements, thereby rendering PA enjoyable, engaging and rewarding [37, 39]. The utilization of gamified scenarios, when designed with sufficient intensity and frequency, has been demonstrated to enhance participation, reduce levels of sedentariness, and promote positive physiological parameters such as heart rate [40]. Despite the limited scope, interventions employing this approach have already shown lasting changes in PA behavior, even long after the intervention has concluded [41, 42].

To effectively support such socially and play-driven engagement in PA, the surrounding environment must also offer opportunities for active participation. In this regard, recent research has increasingly incorporated the concept of *Walkability*, a term that reflects how urban planning and pedestrian-friendly design can facilitate and encourage daily walking behavior [6].

### Walkability and the walkability index

Definitions of *Walkability* vary according to the respective field of research [43]. In the original sense of urban design and planning, *Walkability* refers to the ease with which people can walk in an area. It focuses on the built environment and includes a “set of capacities of any given neighborhood that is embodied in urban morphology in three main ways” [44] including density, functional mix and access networks. These measures can be used to design walkable, transit-oriented neighborhoods, which in turn can increase levels of primarily purpose-related walking or cycling to work or other places, resulting in positive outcomes for PA and public health [44, 45].

From a public health point of view, a walkable area can be defined as a PA-supporting are [44]. With the adoption of this concept by researchers and practitioners in PA and public health, its scope expanded to include walking for both transportation and recreation, as well as other forms of PA such as biking [46]. Consequently, *Walkability* now extends beyond its literal sense of walking alone. Importantly, the influence of built-environment factors varies depending on the manner of walking. Whilst an enhancement in street connectivity, destination accessibility and transit provision is conducive to utilitarian walking, elements such as street greenness, which enhance the aesthetic appeal, safety and comfort, have a more significant influence on recreational walking [47, 48]. Today, the term *Walkability* has a role to play in areas that go beyond urban planning and design, such as public health, climate change and social equality. In this sense, high *Walkability* can have a major impact on increasing residents´ PA and wellbeing, improving air quality, promoting social inclusion and providing pleasurable leisure spaces [49, 50].

One way to assess *Walkability* in a city is to measure a so-called *Walkability Index* which can be used for planning a health promoting and sustainable mobility in the city [51–54]. There are a number of approaches to this issue, each of which results in a slightly different index. However, the majority of these approaches are fundamentally based on the following parameters: density, land use mix, street connectivity and distance to facilities [50, 55] These parameters are measured using Geographical Information Systems (GIS) and aggregated into a single *Walkability Index* [51]. While these components together describe a city’s overall walkability, previous studies have shown that they may affect PA in different ways. For example, a high land use mix can increase accessibility but may also be associated with greater traffic volumes, which could reduce the perceived safety and attractiveness for active school travel. This underlines the importance of examining individual walkability components separately, as discussed later in this manuscript.

Concluding, the *Walkability Index* is based on the narrow incorporation of the parameters of density, land use mix, street connectivity, and distance to facilities according to the field of urban planning. Furthermore, the *Walkability Index* does not consider subjective facets and perceptions of different target groups which may play a decisive role in PA behavior – such as e.g. socio-cultural offers, the accessibility of green space and safety aspects [27, 49, 56–58]. This particularly limits research on children´s and adolescents’ environments and raises the question of whether classic urban design and planning measures, such as the objective *Walkability Index*, can be used in public health research to explain issues such as child and adolescent mobility, and even to inform decisions on PA promotion.

### Problem and hypothesis

Evidence shows a correlation between highly walkable areas and higher levels of active transportation among resident children and adolescents [10]. However, other existing studies have produced mixed results, with some revealing a link between a greater *Walkability* and a decrease in PA [59]. A systematic review found that eight out of 13 studies reported a positive association between higher *Walkability*, resulting from a diverse land use mix and street connectivity, and more active lifestyles. However, five studies did not support this [27]. Laxer and Janssen [60] concluded in their article, that *Walkability* as well as road and park space density, are associated with youth inactivity, a finding supported by Janssen and King [61]. They stated that walkable neighborhood designs may impede PA in children, adolescents, and neighborhoods, as *Walkability* was not associated with active travel to school and was negatively associated with free play. These mixed findings reveal *Walkability* to be a nuanced concept. While some neighborhoods can be categorized as highly walkable due to high street connectivity, they also tend to have more traffic, air pollution and crime as a result of being denser areas with more destinations and people.

Bucksch et al. [62] examined differences between rural and urban areas: Indices for *Walkability* do not seem to be associated with objectively measured PA in either area. Despite higher indices for walkable areas in urban environments, children and adolescents from rural areas walked more for transportation purposes [62]. These findings show that a higher *Walkability Index* does not equate to more favorable PA or health scores, which is also underscored by further research [63]. What accounts for this heterogeneity is mainly incongruities in methodology regarding *Walkability*, but also in defining and measuring PA-related behavior [27].

The involvement of children and adolescents into planning and evaluating processes is a scarce practice and it is difficult to transfer results of studies with adult samples to children and adolescents [30, 64, 65]. They are less autonomous in their decision making and their mobility is mainly limited to their individual neighborhood. Thus, their immediate environment might have a greater effect on their PA compared to adults [30]. Objective measures of *Walkability* such as the *Walkability Index* are mostly constructed from an adult perspective and do not take into account the subjective perceptions of children and adolescents. As a result, no final conclusion can be drawn at this time as to whether the objectively measured *Walkability Index* is coherently related to PA in children and adolescents [27].

This paper therefore examines the association between objective *Walkability* and the activity of children and adolescents in the context of a large-scale intervention in the urban area of the city of Munich in Germany. It is to be expected that this study will provide an answer to the following question: Is there a statistically significant and practically relevant association between the *Walkability Index* of specific urban locations and the levels of PA of children and adolescents carried out there?

## Method

As noted above, it is unclear whether classical urban design and planning measures can be used in public health research to explain issues such as the PA of children and adolescents, or even to inform PA promotion interventions. The “Kreuz & Quer” (“Criss-Cross”) (K&Q) intervention is a project combining measures of urban planning and public health research, implemented by the mobility department of the City of Munich and the XXXUniversity XY in seven city districts [66].

### Data composition

The key variables for investigating the research question are *Activity* (indicator for PA) and *Walkability* (measured by the *Walkability Index*). *Activity* data was obtained from the K&Q intervention database. The measure is device-based and objectively recorded, reflecting the number of digitally logged interactions at physical checkpoints distributed across the intervention area. The *Walkability Index* was provided by the department for climate protection and environment of the city of Munich [53]. In addition, the city district from which the data originated (*District*), the time of year at which it was collected (*Season*) and the day of the respective wave of K&Q from which the data originated (*Day*) have been documented as additional variables. Data sources for *Activity* and *Walkability* are described in more detail below.

#### *Activity*: physical activity data of the intervention

**Fig. 1:**
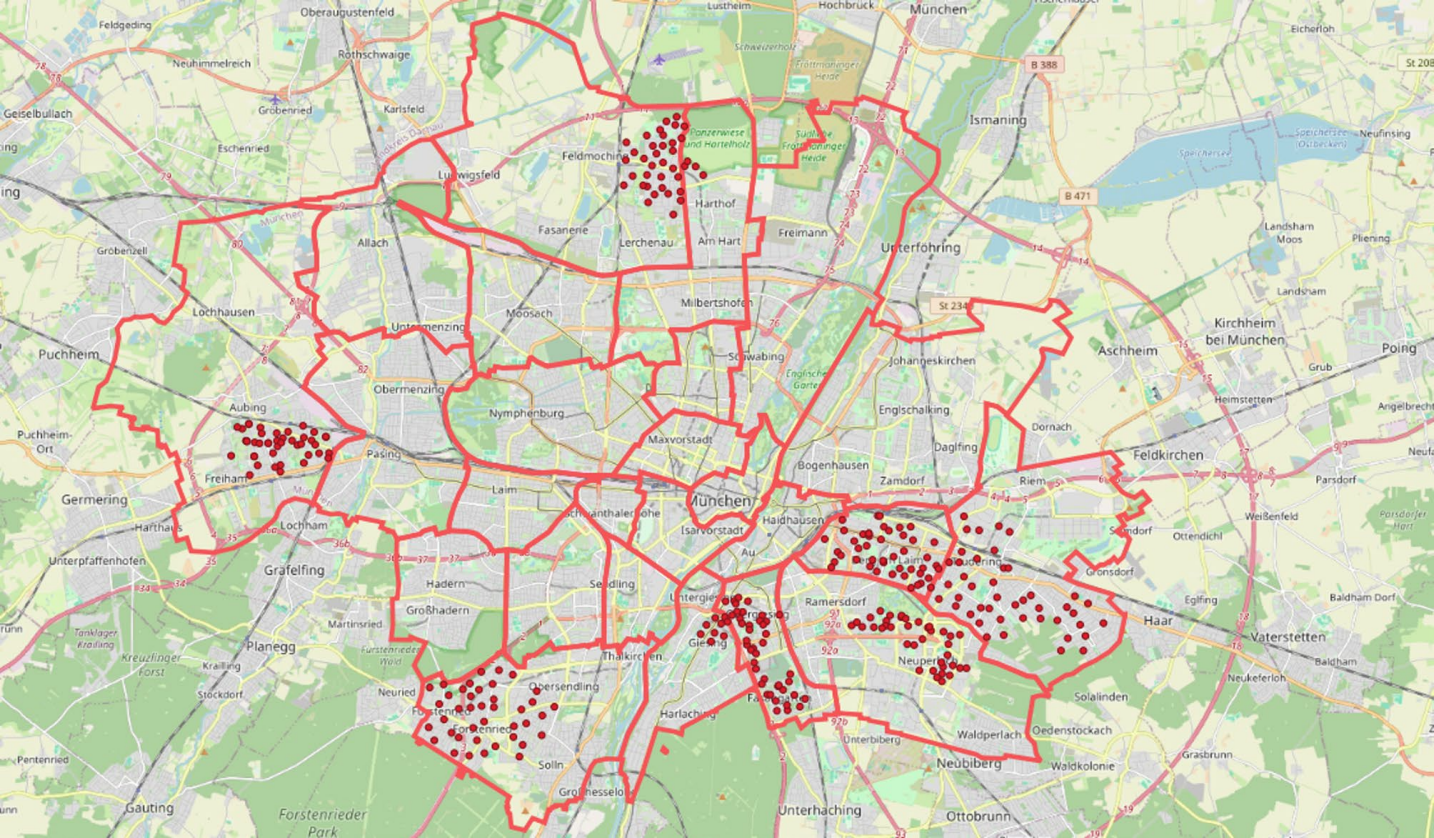
Munich; the red dots represent the box locations, the red lines represent the district borders

The K&Q project was initiated in 2019 by the mobility counsellor of the city of Munich. The intervention was carried out in three waves per year for four to six weeks in different city districts each. It was a gamified urban intervention that used a digital scavenger hunt-like format, encouraging resident children and adolescents to explore their neighborhoods by locating and scanning physical checkpoints, in form of electronic boxes, to collect points. The participants collected points as individuals or teams, such as school teams. In each chosen neighborhood, several different boxes were installed on accessible objects on the streets, for example streetlamps, for a certain period of time. The electronic boxes were regularly distributed in the seven neighborhoods at distances of about 500 m. The intervention will be gradually implemented in all districts of Munich over time. The seven districts considered in this analysis are therefore not a random sample, but instead represent seven districts in immediate succession. Figure 1 visualizes the box locations in each of those districts on a map of Munich. The mean area size of neighborhoods where the intervention took place is 12,7 km^2^ (± 6,7).

Participants received a physical game card that can be scanned at the respective boxes. Each scan was rewarded with points. Participants could walk, bike or scooter from one box to another to scan their cards for collecting points and kilometers over the entire period of the campaign. Higher points were awarded for scanning more different boxes without a major break. The intervention incorporated gamification elements to keep participants motivated. These elements included daily ranking updates on the homepage, instant audio feedback after scanning a box, or the chance to win attractions such as a bouncy castle, a photo box or face painting for the final award ceremony. Participants were recruited through information campaigns at the schools via posters and flyers by the mobility council of the City of Munich. The legal guardians of underage participants had to give written informed consent to the use of their children’s data in order to obtain a game card for their children.

For the purposes of this analysis, each scan at one of these electronic boxes is counted as one *Activity*, which is the indicator for PA. The more often participants have visited a particular box, the higher the *Activity* of that box. Although the number of box visits does not directly quantify duration or intensity of PA, it represents a valid behavioral indicator of active mobility within the intervention context. Each scan required participants to travel actively to a physical location, thereby reflecting real-world movement between spatial points in the neighborhood. Similar gamified public health interventions, such as *Beat the Street* [41, 42], have used comparable interaction counts as proxies for physical activity and community engagement. While the measure cannot differentiate between light, moderate, and vigorous activity, it reliably captures the frequency and spatial distribution of active trips. The “Activity” indicator therefore serves as a pragmatic, objective, and non-reactive proxy for children’s mobility and engagement in neighborhood-based movement.

#### *Walkability*: an index for Munich

The three-dimensional *Walkability Index* employed in this study builds on the original framework by Frank et al. [51], who proposed four main dimensions: density, land use mix, street connectivity, and floor area ratio (FAR). In more recent European applications, however, the FAR component has often been omitted due to limited data availability and its lower contextual relevance in compact European cities. Instead, simplified indices based on three dimensions of population density, land use mix (entropy), and street connectivity have become standard practice [67].

Munich is divided into 25 city districts. Each of these districts is further subdivided into smaller neighborhood units for statistical and administrative purposes. The *Walkability Index* is calculated on the smallest-scale level in so called “Stadtbezirksviertel” (SBV). The amount of SBVs (475 in total for Munich) per district varies depending on the size and structure of the district. The *Walkability Index* is calculated from *population density* (population per km^2^, standardized to z-score), *connectivity* (number of crossroads per km^2^, standardized to z-score) and *entropy* (measure for a balance of the distribution of living space, industry, culture, and administration in a neighborhood, standardized to z-score). All standardized z-scores from the three elements are combined to the final *Walkability Index* for each SBV [53]. The z-score standardization of the three components was calculated across all 475 SBVs of Munich to ensure city-wide comparability of walkability scores. This means that each SBV’s value represents its deviation from the overall mean of all SBV within the city, rather than only those included in the intervention areas. This results in scores from − 9 to 13.1, whereupon higher values indicate a higher *Walkability*. Based on this small-scale measurement of the *Walkability Index* for the SBVs, every box of K&Q can be assigned to their own individual *Walkability Index* score.

*Walkability* is an indicator of the attractiveness of walking, which makes it interesting to see whether the two indicators of *Activity* and *Walkability* are associated and whether a higher attractiveness of walking (*Walkability*) leads to more scans of the corresponding box (*Activity*).

### Data Preparation and analysis

To test the association between *Activity* and *Walkability*, a linear mixed model (LMM) approach was implemented for the main analysis. Fixed effects correspond to the relation between the main predictor variables and the dependent variable (*Activity*). In this case, *Walkability* was defined as fixed effect [68]. The effects, which are not argued to be the association that is studied but could have an influence on the results, are referred to as random effects. In this case, *District*, *Season* and *Day* were therefore included as random effects with regard to their associations with *Activity*. In summary, this means that primarily the association between the dependent variable (*Activity*) and the fixed effect (*Walkability*) was examined, as well as the associations with the random effects were also considered (*District*,* Season*,* Day*).

The initial visual inspection of the collected data was able to rule out heteroscedasticity. Based on the qualitative evaluation of the QQ plot, however, a positive skewness and thus a violation of the normal distribution had to be assumed. A log transformation was therefore carried out, which significantly improved the model quality. The comparison of different model variants attested the best fit to the more complex model described above (Activity ~ Walkability + (1 | District) + (1 | Season) + (1 | Day) in comparison with simpler model variants or model variants with interaction effects measured by AIC and BIC indicators [68]. Due to the model and variable structure, multicollinearity was not tested. The preparation of the data and the statistical analyses were conducted with the software R [69] and the editor R-Studio.

## Results

### Descriptive results

To further describe the districts included for the intervention the respective population size under 18 years and the number of households with children was calculated from the monitoring of the social department of the City of Munich. Table 1 provides a comprehensive overview of the numbers for each district, categorized according to the year in which the respective round of K&Q took place. Table 2 provides an overview of the main variables for all seven districts, including the season, number of participants with mean age and standard deviation, duration of each intervention wave, number of boxes, summed number of activity, and the mean value for the *Walkability Index* with range. For a descriptive evaluation of the success of K&Q, see also [66]. The age of the participants was documented with the information being provided by participants who had completed the registration process in a satisfactory manner (58% of all participants). The mean age for all participants was 9.7 years. The single interventions in the neighborhoods lasted from 29 to 47 days (mean = 41.8) and a total of 300 boxes existed during the whole period. The highest number for *Activity* can be seen in district 2, with a total of 173,997 box scans. Table 2 points out that the neighborhoods in districts 2 and 4 have the highest mean value of *Walkability*, the lowest mean value is assigned to district 3. According to the present data the mean *Walkability* indices for the respective neighborhoods range from − 3 (district 1) to 8.34 (district 2).

**Table 1:** Characteristics of the seven districts including the respective year the intervention took place, the underaged population and the number of households with children

District	Year	Population < 18	Households with children
1	2019	3135	1856
2	2022	9362	6046
3	2022	9969	6114
4	2022	8750	5244
5	2023	10174	6056
6	2023	13432	8192
7	2023	7675	4580
Mean		8928,1	5441,1

**Table 2:** Descriptive characteristic values (sums and means) for the central variables

District	Season	Participants *N*	Mean age ± SD	Days	Boxes *N*	Activity sum	Walkability mean (range)
1	Fall	961	8,5 ± 2,1	45	43	56,099	1.04 (−3; 4.36)
2	Spring	1946	8,4 ± 2	47	51	173,997	3.63 (0.67; 8.34)
3	Summer	1071	10,3 ± 1,9	29	50	51,937	0.23 (−3; 3.08)
4	Fall	876	7,9 ± 2	47	38	56,164	3.14 (0.67; 8.34
5	Spring	1827	9,7 ± 2	42	38	114,556	1.14 (−2.4; 3.51)
6	Summer	1778	10,1 ± 1,7	36	40	121,929	1.04 (−1.9; 4.51)
7	Fall	1487	9,6 ± 1,9	47	40	51,033	2.82 (−1; 4.85)
Mean		1407.4	9.7	41.8	42.9	89387.9	1.72 (−3; 8.34)

### Statistical results

#### Associations between *Activity* and *Walkability*

The inferential statistical analysis using a LMM provides both indications of the extent to which *Activity* and *Walkability* are associated with each other, as well as how large the proportion of additional variables is in explaining the calculated variance.

**Fig. 2:**
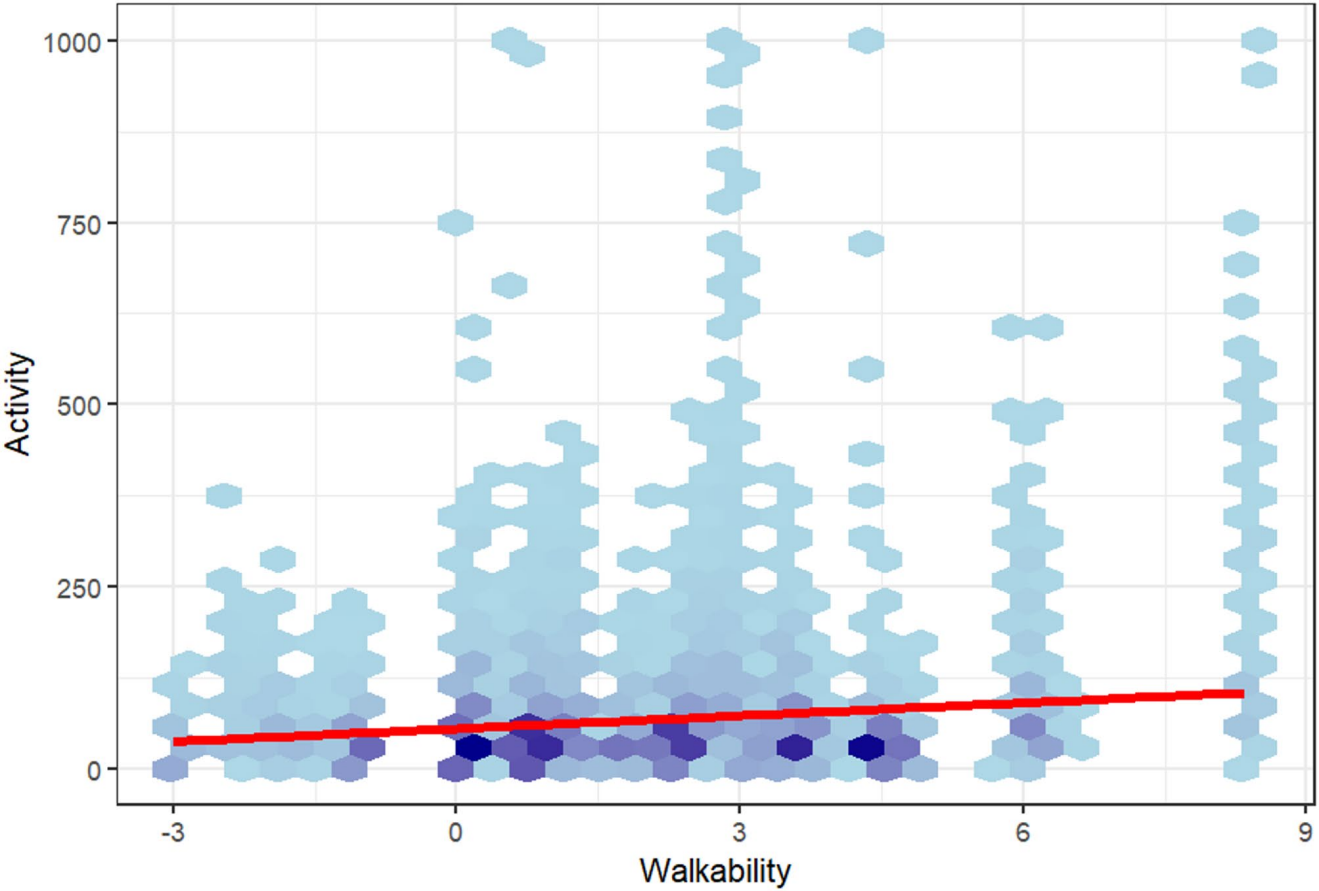
Hexbinplot showing the association between *Activity* (cumulated for each box over the whole corresponding wave) and existing *Walkability* index scores of the box locations (a darker color of the hexagon indicates a higher density, i.e. a concentration of associating *Activity* and *Walkability* values in this area)

As shown in Table 3, *Activity* and *Walkability* show a significant association with a p-value of < 0.001. Due to the very low standard error, the estimate of 0.0142 can be certified as highly reliable. Given the log-transformation of the dependent variable, the fixed-effect coefficient (β = 0.0142) indicates an average increase of approximately 1.4% in Activity per one-unit increase in Walkability. The Hexbinplot in Fig. 2 illustrates the positive association between *Activity* and *Walkability*. Based on this data, an accumulation of *Walkability Indices* in the range between 0 and 4.5 can be observed. Areas with very low *Walkability Indices* also display very low *Activity* scores.

**Table 3:** Fixed effect: statistical key figures on the association between *Walkability* and the dependent variable of *Activity*

	Estimate	Standard Error	*p*-value
*Walkability*	0.0142	0.00134	< 0.001

#### Random effects of the model

Aside from this statistically significant association between *Activity* and *Walkability*, additional factors account for part of the observed variation in the data. Table 4 serves as an overview for the explained variance of the random effects *District*,* Season* and *Day* in the previously analyzed model.

**Table 4:** Random effects: statistical key figures on the proportion of the variance of the model explained by the variables *District*, *Season* and *Day*, as well as the variance not explained by the model (*Residual*)

		Variance
*District*	(Intercept)	0.008707
*Season*	(Intercept)	0.003888
*Day*	(Intercept)	0.005699
*Residual*		0.059434

There is a certain difference in the *Activity* scores that can be explained based on the respective *District* in which the respective wave of K&Q took place (proportion of the explanation of the total variance of the model: 0.008). It should be noted that the variance components of the random effects are descriptive in nature. Their absolute values are not directly interpretable and mainly serve to indicate relative variation compared to a null model without covariates. Thus, these estimates illustrate contextual clustering effects rather than explained variance in a strict statistical sense. As shown in Fig. 5, districts 3, 4 and 7 stand out with a comparatively higher level of *Activity* based on the average values. The comparatively smallest effect can be attributed to the *Season* factor (0.003), whereby those rounds of K&Q that took place in spring and summer generated a higher level of *Activity* (see Fig. 4). The *Day* factor explains 0.005 of the variance. As illustrated in Fig. 5, a negative association can be assumed here, meaning that the longer the respective round already lasts, the lower the *Activity* on that day. The comparably extremely high value of variance explanation for *Residual* in comparison to these factors implies that the model in this form cannot explain a total of 0.059 of the variance.

**Fig. 3:**
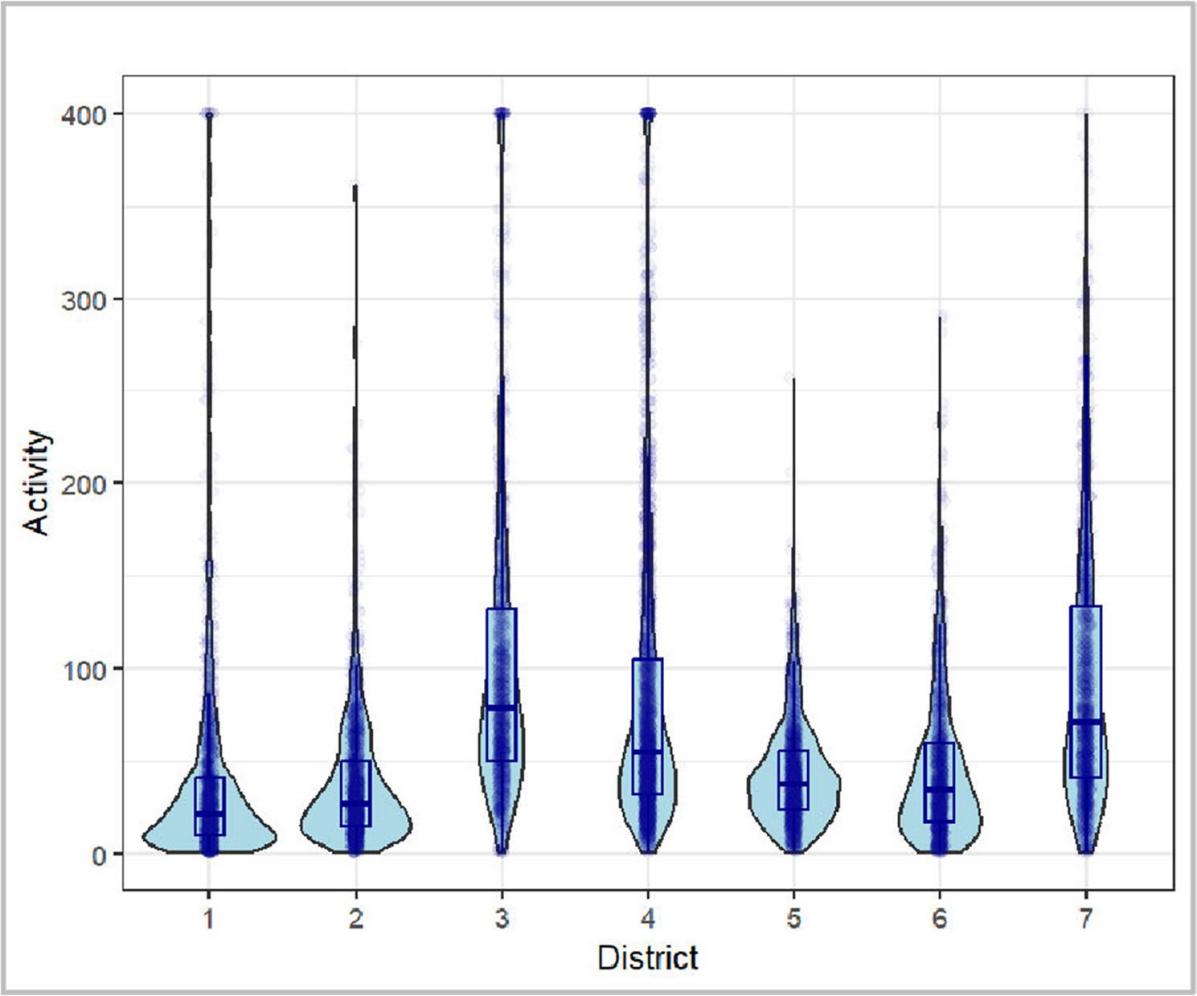
Violin-boxplot comparing the cumulative *Activity* per day and box in the different *Districts*

**Fig. 4:**
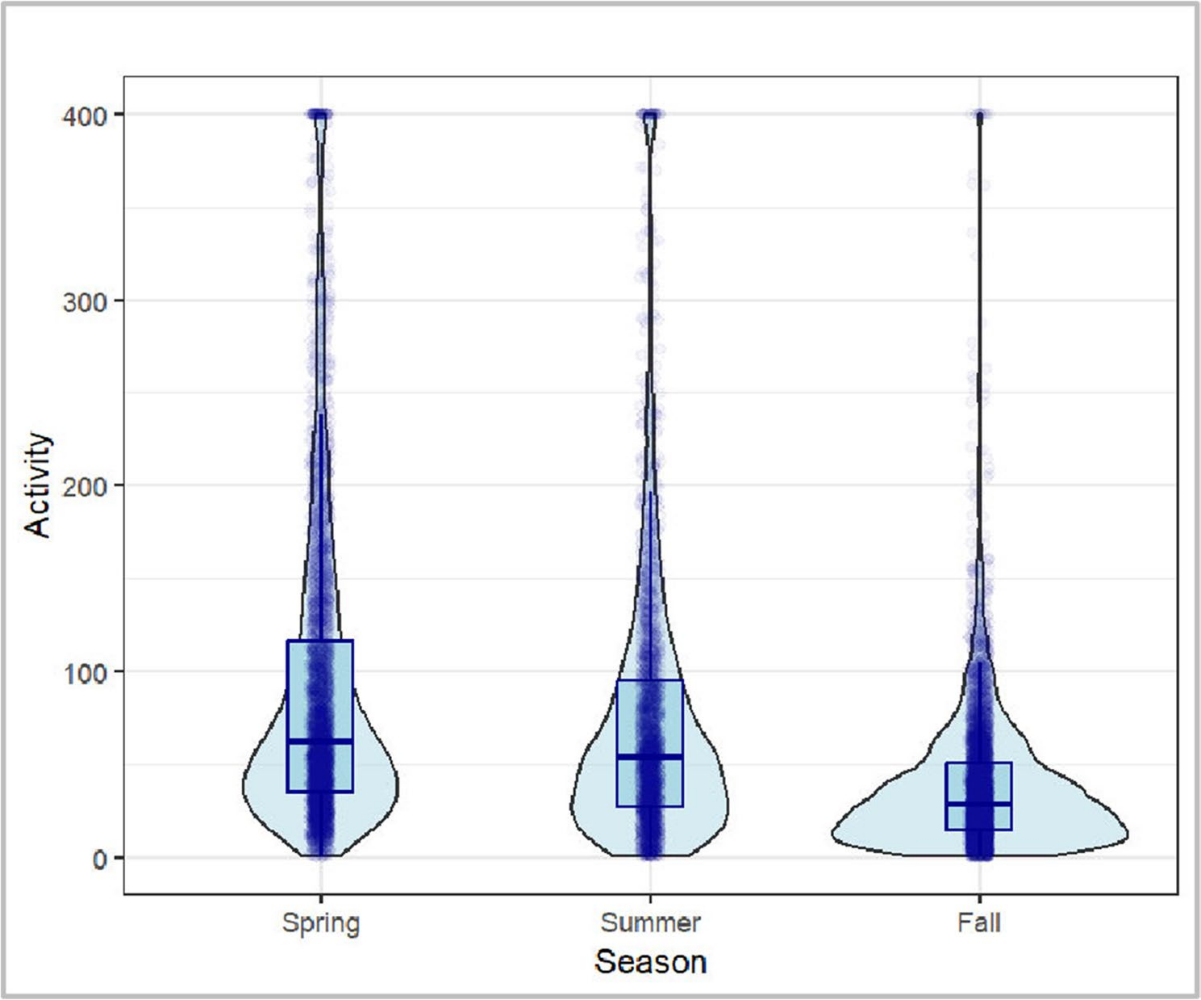
Violin-boxplot comparing the cumulative *Activity* per day and box in the different *Seasons*

**Fig. 5:**
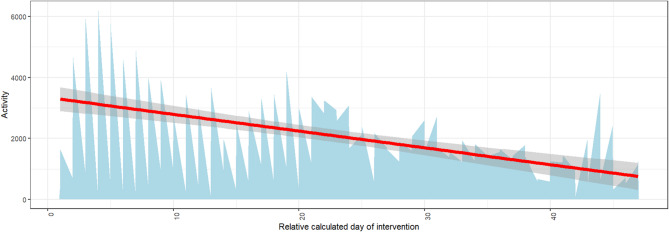
*Activity* per day over time in the sum of all intervention rounds (due to the different lengths of the rounds, all were normalized to the maximum length; the daily data plotted on the x-axis are therefore to be understood as relative, with the first day of each round on the left and each last day of each round on the right). *Activity* represents the number of scans at all boxes

## Discussion

The present study investigates the association between *Walkability* by using the *Walkability Index* of the City of Munich, and an indicator for children´s and adolescents´ *Activity* collected as part of an intervention to promote urban PA behavior. Influences of the respective *District*, *Season* and *Day* were examined, too. The results show that the *Walkability Index* is a significant positive predictor of *Activity* as an indicator for PA in this study. Random effects capture part of the contextual variation, although a substantial proportion remains unexplained.

### Association of *Walkability* and *Activity*

The results show a significant association between *Activity* and *Walkability.* It seems that factors resulting in a high *Walkability Index* also play a decisive role for the *Activity* of children and adolescents. According to the elements the index comprises following aspects could explain the observed association. It should be noted here that the discussion of *density*, *entropy*, and *connectivity* is based on theoretical reasoning and previous literature. In the present analysis, these components were aggregated into a single composite index provided by the City of Munich. As the underlying raw data and component scores were not accessible to the authors, it was not possible to include the individual components as separate predictors in the statistical models.

The extended concept of *Walkability* also includes social determinants, such as the number of physically active people in a neighborhood influencing the PA level of people living in this area [70]. Population *density* therefore could be relevant for children as a higher *density* means more people in one area which could be related to more playmates and social interaction. The fact that *density* is conducive to a higher level of social activity for the residents of the area is also explained in the article by Fina et al. [29]. Another possible explanation for higher PA due to higher population density is that more retail services and facilities in these areas increase the number of potential destinations within walking or cycling distance, encouraging PA [71]. A higher *density* could also mean that more people, including children, live in the vicinity of the box locations, so that more people walk past the boxes and *Activity* increases.

The second objective factor for *Walkability* is land use mix, which is represented by the entropy index. The element of *entropy* includes land use types as *sport* and *recreation* which indeed have a positive influence on PA behavior and enhance children ´s activity [29, 53, 72]. The mixture of *habitation*,* retail* and *recreation* enables children to explore a diverse environment and to have more offers at their disposal. A high land use mix contributes to the livability of a neighborhood and a more appealing walking environment, promoting healthier lifestyles [73]. Consequently, high entropy is indicative of a diverse range of amenities and services available within a given neighborhood.

A higher *connectivity* is related with shorter distances being beneficial for children as they can walk or bike to school, friends, or sports more easily. It also means having more options to choose from and, ultimately, deciding to opt for a more attractive route. On the other hand, a higher street connectivity may be related to fewer cul-de-sacs and thus be high-traffic areas which hinder children from outdoor PA [74]. In the context of the K&Q intervention, high street connectivity and traffic might discourage children from crossing roads. Even if they see a box on the other side of the road, they cannot reach it to scan it, resulting in a decrease in *Activity*. This is also linked to the presence of safety features such as pedestrian crossings or traffic lights [75]. Those safety aspects are not included in the *Walkability Index*, but do play a decisive role for the PA behavior of children and adolescents and should be included in future research [76, 77]. The extent to which greater connectivity is ultimately beneficial or detrimental remains equivocal.

It has thus been demonstrated that *Activity* and *Walkability* are indeed associated. This could, in principle, be explained by a closer analysis of the elements that make up the index. This is despite the fact that it has been questioned whether such an objective index is at all suitable without the subjective perspective of children and adolescents. The *Walkability Index* for Munich is made up of three factors which all can have stronger or weaker influences on the respective district and the citizens living there. Despite the positive association, there are diverse additional factors that play a role when analyzing activity patterns completely. Access to recreational facilities, school environments, or parental supervision may be more decisive for youth PA than *Walkability* alone [78]. Therefore, *Walkability* can serve as one but not the only explanatory approach concerning children´s PA behavior in respective neighborhoods, as is evident from the figures relating to the variance explained by the random effects.

### Influencing factors of physical activity

The included random effects *Day*,* Season* and *District* were able to explain a part of the variance. The variable *Day* showed a negative association, meaning that *Activity* decreases over time. The longer the intervention lasts, the less active participants become. This could be due to a better acceptance and more enthusiasm of the participants at the beginning of the intervention, as previous studies have shown that interest in interventions declines over time [79].

An examination of the results for the variable *Season* reveals that the majority of cumulative *Activity* occurs in the spring and subsequent summer, with a comparatively smaller amount occurring in the fall. Temperatures, precipitation as well as day length vary across seasons and might affect PA behavior [80]. Thus, weather conditions play a role as it could have been exceptionally hot in the summer months and worse conditions, like cold or rain, appeared in fall months, refraining children from being more active outside. Especially Spring and Summer are predestined to cause higher PA levels [80–82]. As the results show an influence of seasonal variations on PA, this should be considered in future public health research and the design of interventions to promote PA, as seasonality can be perceived as both a facilitator and a barrier [82]. Interventions could be adapted depending on the season with specific challenges, outdoor- and indoor-activity opportunities or weather adapted events.

The variable *District*, which explains part of the variance in *Activity*, reflects a range of underlying contextual factors influencing PA. These may include geographical, social, and demographic characteristics. Smith et al. [16] highlight that built environment features relevant to PA, such as access to green spaces, perceived safety, infrastructure for active transport, and land use mix, are often unequally distributed across urban districts, particularly along socioeconomic lines. In addition, districts may vary in terms of population composition, cultural norms, availability of recreational facilities, and exposure to traffic or environmental stressors, all of which shape opportunities for and attitudes toward PA. Although intra-district variation exists, district-level clustering likely captures macro-level environmental and social disparities relevant to urban PA behavior. These contextual factors may explain variance not accounted for by objective indicators like the *Walkability Index* alone. As Gemmell et al. [83] point out, neighborhood characteristics influencing outdoor play include playgrounds, sidewalks, destinations, green spaces, traffic levels, social safety, and cohesion. Since these factors can vary substantially between districts, differences in PA behavior are a plausible outcome.

However, a great part of the model variance is not explained by the included fixed or random effects. It is possible that a proportion of the unexplained variance in the model can be attributed to the fact that the index developed by Frank et al. [51] was not originally designed to assess *Walkability* for children and adolescents. It is noteworthy that the attributes may not be aligned with the specific requirements of the individual’s walking needs [84, 85]. Furthermore, it suggests the presence of additional factors which may neither be displayed by objective indices nor theory-based models. One way of approaching these factors is to include the target group and their subjective view of corresponding interventions. As Wang et al. [33] explain in their research, the impact of the built environment on an individual´s PA behavior cannot be fully described by using objective measurement tools. However, research demonstrates that both perceived and objective indicators of *Walkability* are useful to fully grasp influencing factors [32]. A positive example for a subjective approach can be named with the study “Walki-Muc” [30]. The promising measure of the Neighborhood Environment Walkability Scale for Youth (NEWS-Y) questionnaire incorporates the subjective perception of children and adolescents regarding diverse environments. Current research highlights the adaptation of *Walkability* indices with target specific factors. Buck et al. [84] reveal recreational facilities such as playgrounds and public open spaces as important features for PA. Based on that, the researchers developed moveability indices with strong effects on PA in school children. These examples highlight the importance of considering multi-level influences when planning, implementing, and evaluating PA in children and adolescents in their neighborhoods.

### Practical implications in the context of urban planning

Objective indices, such as the *Walkability Index*, have the capability to function as a guide in order to identify environments that may support or hinder PA. It is hard to imagine that areas with extremely low *Walkability Indices* are particularly attractive to children and adolescents and their PA behavior. However, if one aims to promote the PA of a specific population, additional data gathering methods as well as participative approaches should be applied on the development of interventions. The concept of co-creation, being one of many participative approaches, engages and empowers end-users and is proposed to increase behavior intervention adoption, adherence, and effectiveness [86]. Research suggests implementing the subjective perspective of vulnerable groups, in this case children, as they perceive their environment in a different way than adults do [10, 30, 65]. The *Walkability Index* is generally developed for adult mobility patterns, which may not fully capture how children and adolescents use their environment. When addressing children and adolescents, it could be useful to develop child-friendly *Walkability-*criteria consisting of safe, playful, and social factors and combining aspects such as traffic-calmed infrastructure, co-creation processes, and technological incentives. Consequently, the development of a Youth Walkability Index, grounded in the *Walkability Index* and adapted through qualitative and participatory methodologies, could prove more efficacious [30]. Moreover, incorporating additional demographic factors, such as socio-economic status (SES), could facilitate an assessment of their influence on mobility choices and behaviors. Research has yielded inconsistent results regarding the mediating effect of demographics on the association between *Walkability* and PA [87, 88]. Further research is necessary to provide more robust evidence on this matter. In exploring SES-moderated disparities in PA, these findings could be employed in urban planning to address inequalities [89].

### Strengths and limitations of this study

The combinability of a *Walkability Index* officially surveyed in a city with millions of inhabitants, which requires a large number of parameters, often only available to the local authority, and a large-scale study on PA promotion with very large expenditure in terms of time and money, is scarce and offers unprecedented insights. The present study examined data from seven city districts, accounting for approximately 62,500 children and adolescents under the age of 18 living in the seven districts in question. Of these, 9,852 participated in the K&Q, representing a proportion of 15.7%. Key strengths include the use of sound data sources and analytical methods to support the findings, practical outcomes, and the ability to compare results to those of other studies and cities. Specifically, *Walkability* and an indicator for PA were measured objectively. This approach ensured that no recall bias, social desirability, or similar factors influenced the results, as is typical of similar studies.

However, a key limitation concerns the non-standardized nature of the “Activity” indicator. Although each box interaction represents an objectively verifiable episode of active movement, the measure has not yet been formally validated against accelerometer or pedometer data. Future research should aim to triangulate such gamified activity metrics with reference methods to assess their convergent validity and reliability. However, previous interventions using similar designs (e.g. *Beat the Street*) demonstrated consistent associations between game interactions and accelerometer-based increases in PA [41, 42], suggesting that this proxy captures meaningful behavioral change in real-world settings.

As stated above, district-level socioeconomic indicators such as deprivation or welfare rates (e.g., SGB-II quota) were not included in the present analysis. Although such data might provide contextual information about neighborhood characteristics, they cannot reliably capture the individual socioeconomic background of participating children and adolescents. Since the association between neighborhood-level deprivation and individual-level SES is often inconsistent, inclusion of these variables could introduce additional uncertainty rather than clarification. Nevertheless, future research could explore this dimension once sufficiently granular and harmonized data become available.

Furthermore, large parts of the variance cannot be explained by the variables collected. However, due to the practically unmanageable number of additional variables, a qualitative flanking would be particularly valuable. In this way, quantitative calculations could be combined with qualitative explanatory backgrounds to create a holistic picture. The combination of quantitative and qualitative elements is particularly advantageous for complex intervention planning and provides a better understanding of research problems [90]. This mixed-methods design could increase the gain in knowledge and promote the inclusion of target groups [91].

Another limitation is that the results show associations rather than cause-and-effect relationships. In order to create a longitudinal study that can provide a higher level of evidence for causal relationships, children’s PA levels and movement patterns could be measured before and during an intervention. This would provide additional interpretative approaches in this context, as well as additional variables to be collected.

In addition, the analysis focused exclusively on macro-level measures of the built environment, such as population density, land use mix, and connectivity. Future research should complement these structural indicators with micro-level environmental features, including the quality of walking infrastructure, presence of greenery, and neighborhood aesthetics, which are also known to influence PA behaviors.

## Conclusions

The *Walkability Index* is a resource-saving, objective method that was applied in this study and proved to be associated with PA in children and adolescents. The index could thus be utilized in PA research for the purpose of intervention planning. Additionally, it can be employed in the field of urban planning, aiming at creating a PA-promoting environment for children and adolescents in an urban context. Differences in PA patterns between districts, seasons, and days are relatively small, but should be taken into account in future planning efforts. Despite that, a considerable amount of variance remains unclear, suggesting that important influencing factors have yet to be identified. Therefore, the unique needs and subjective perceptions of children and adolescents regarding *Walkability* could be given greater consideration in future urban planning processes to better understand their needs and reveal additional influencing factors. Integrating these perspectives can help create environments that are not only structurally conducive to walking but also perceived as safe and inviting by younger populations. Future urban planning strategies should embrace a more tailored, evidence-based approach that ensures sustainable, health-promoting, and inclusive cities for all.

## Data Availability

The datasets used and/or analysed during the current study available from the corresponding author on reasonable request.
